# Supplementary material for: Extremophiles in Soil Communities of Former Copper Mining Sites of the East Harz Region (Germany) Reflected by Re-Analyzed 16S rRNA Data
Source: Microorganisms. 2021 Jun 30;9(7):1422. doi: 10.3390/microorganisms9071422 (PMC8305195; doi:10.3390/microorganisms9071422)
Supplement: Supplementary file 1 [file microorganisms-09-01422-s001.zip › microorganisms-1143772-supplementary.pdf]

microorganisms

MDPI

Supplementary material

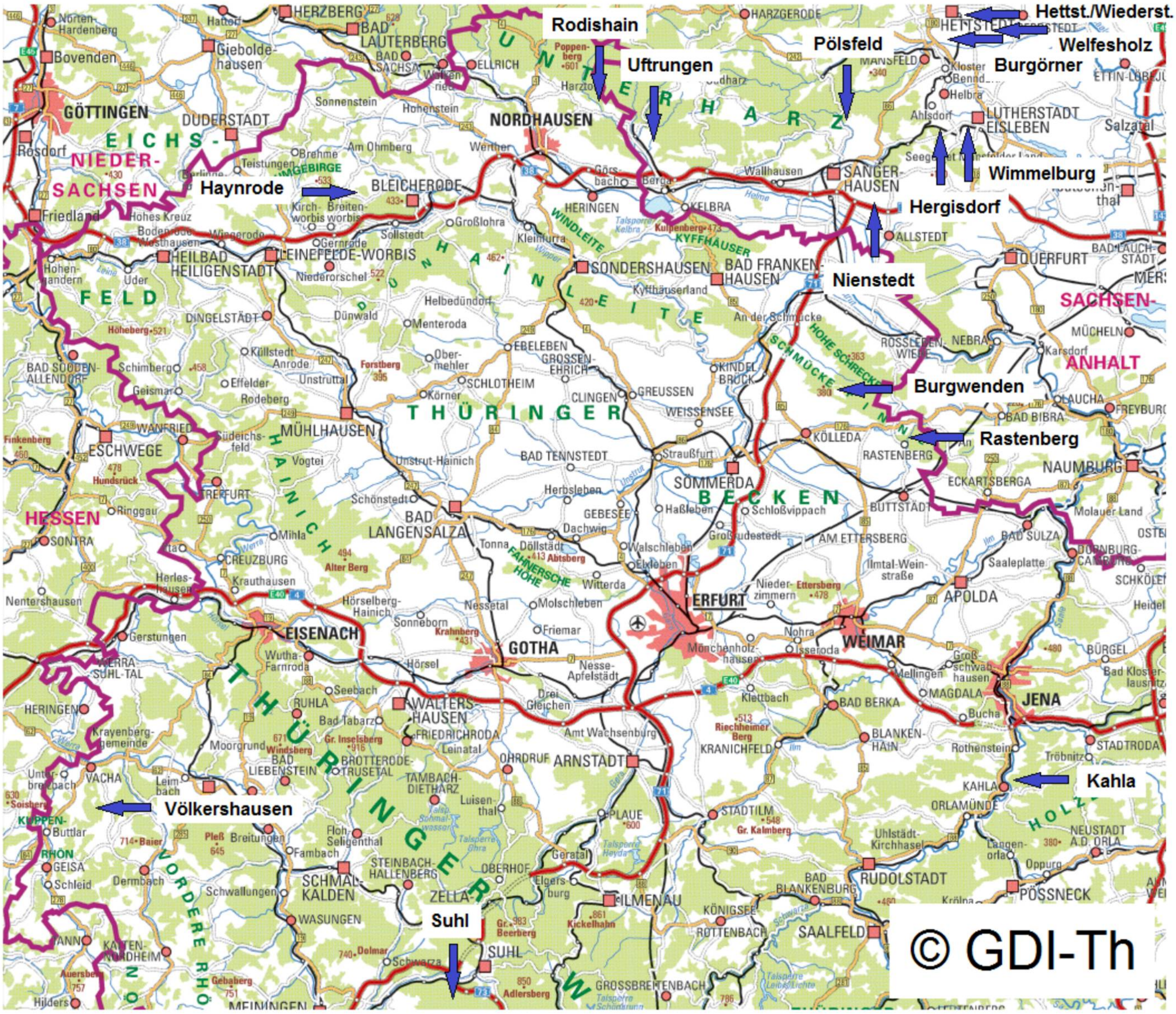

Figure S1. Origin of samples ((C) GDI-Th).

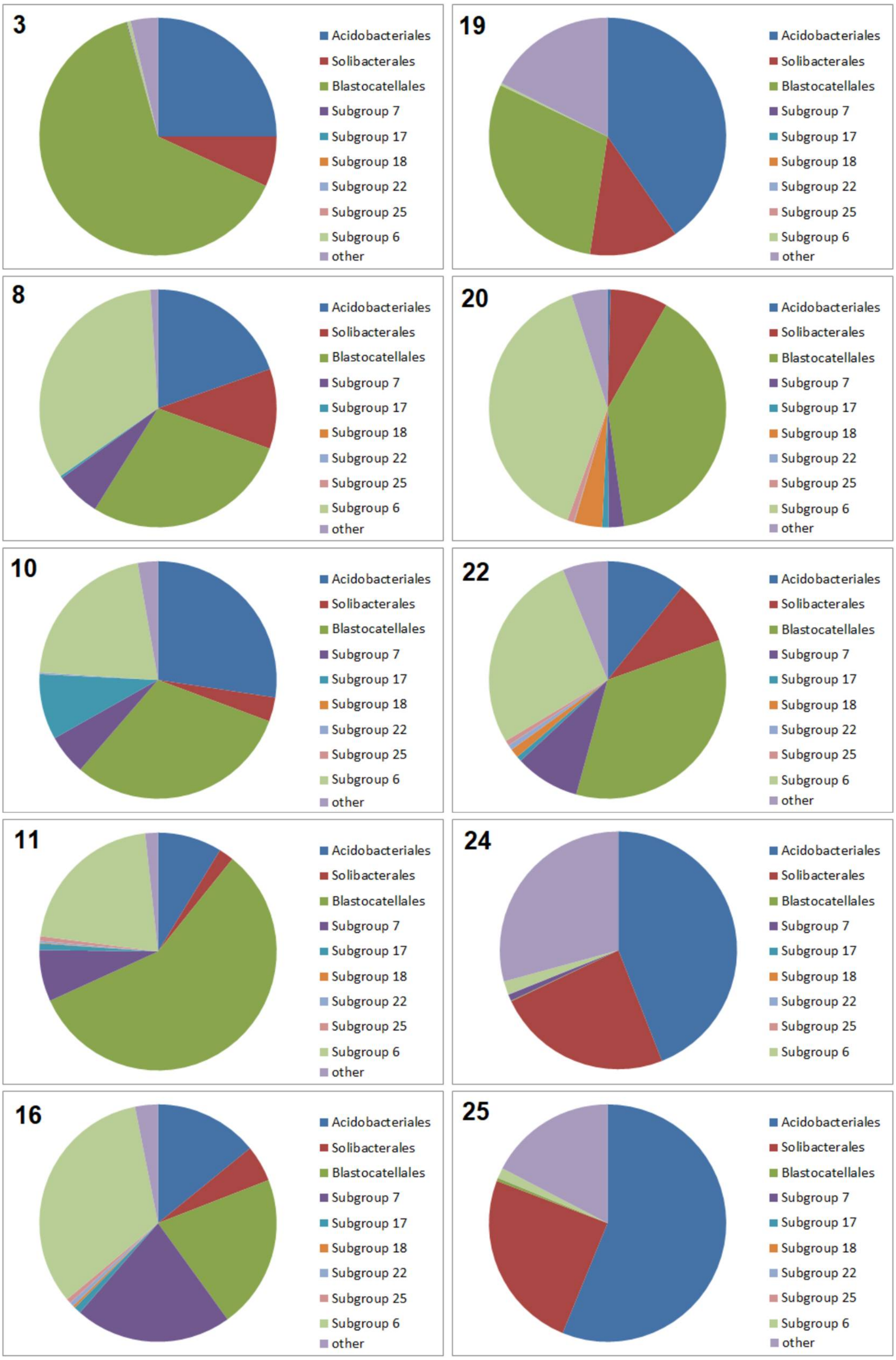

Figure S2. Distribution of abundant orders of Acidobacteria (percentages of orders in the class) in 5 selected samples from ancient mining areas (No. 3 Wimmelburg, slag deposit, pH 5.57), 8 (Welfesholz, group C, pH 8.78), 10 (Burgörner, group C, pH 8.85), 11 (Burgörner, group C, pH 8.28), 16 (Uftrungen, Group A, pH 6.23), from industrial mine dump (No. 19, Nienstedt, pH 7.66, extraordinary high electrical conductivity) and four comparative samples (limestone substrate: No. 20 and 22 (group F), acid soil: No. 24, 25 (group G)).

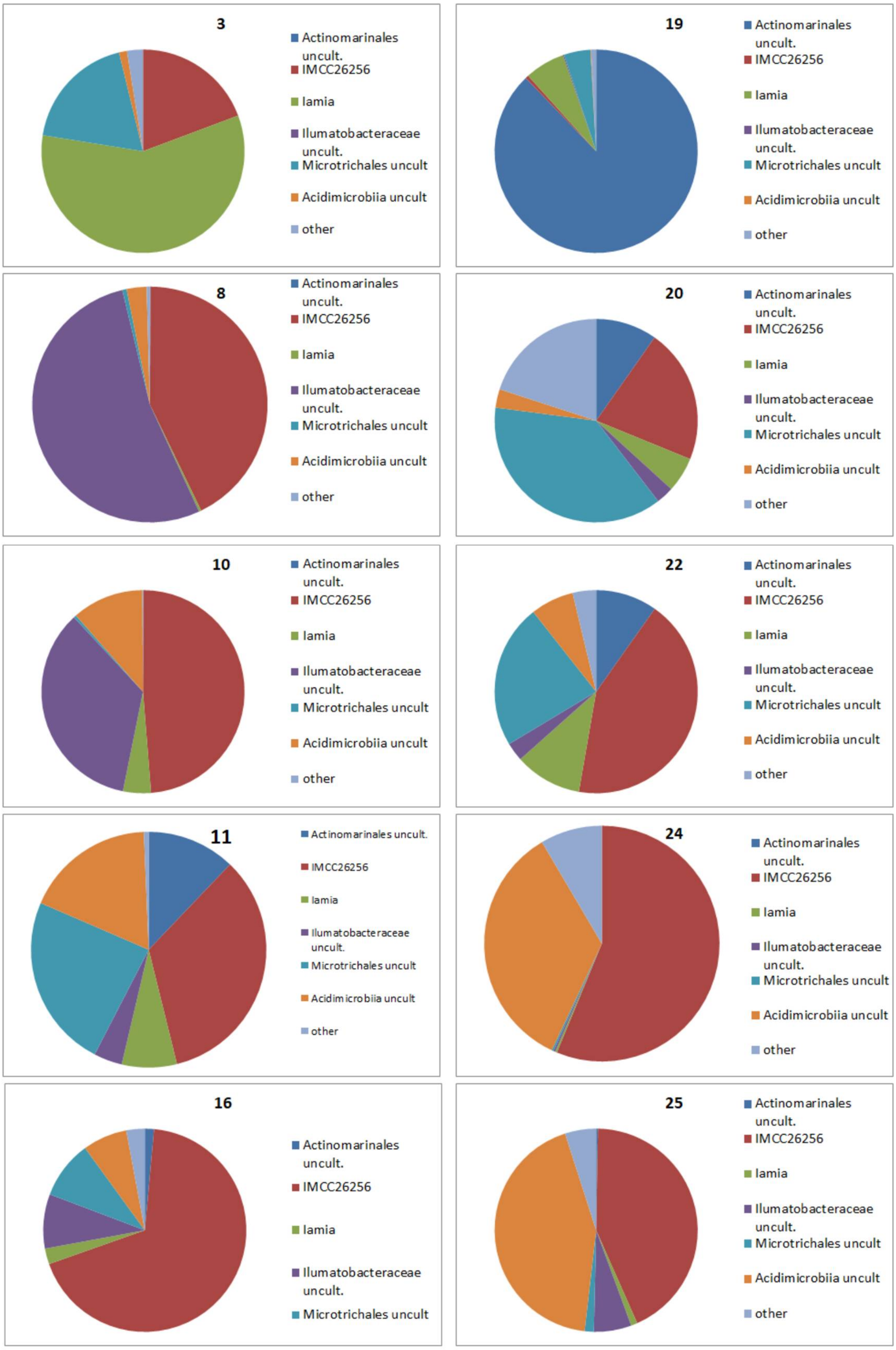

Figure S3. Distribution of abundant OTUs of Acidimicrobiia (percentage of classes in the phylum Actinobacteria) in 5 selected samples from ancient mining areas (No. 3 Wimmelburg, slag deposit, pH 5.57), 8 (Welfesholz, group C, pH 8.78), 10 (Burgörner, group C, pH 8.85), 11 (Burgörner, group C, pH 8.28), 16 (Uftrungen, Group A, pH 6.23), from industrial mine dump (No. 19, Nienstedt, pH 7.66, extraordinary high electrical conductivity) and four comparative samples (limestone substrate: No. 20 and 22 (group F), acid soil: No. 24, 25 (group G)).

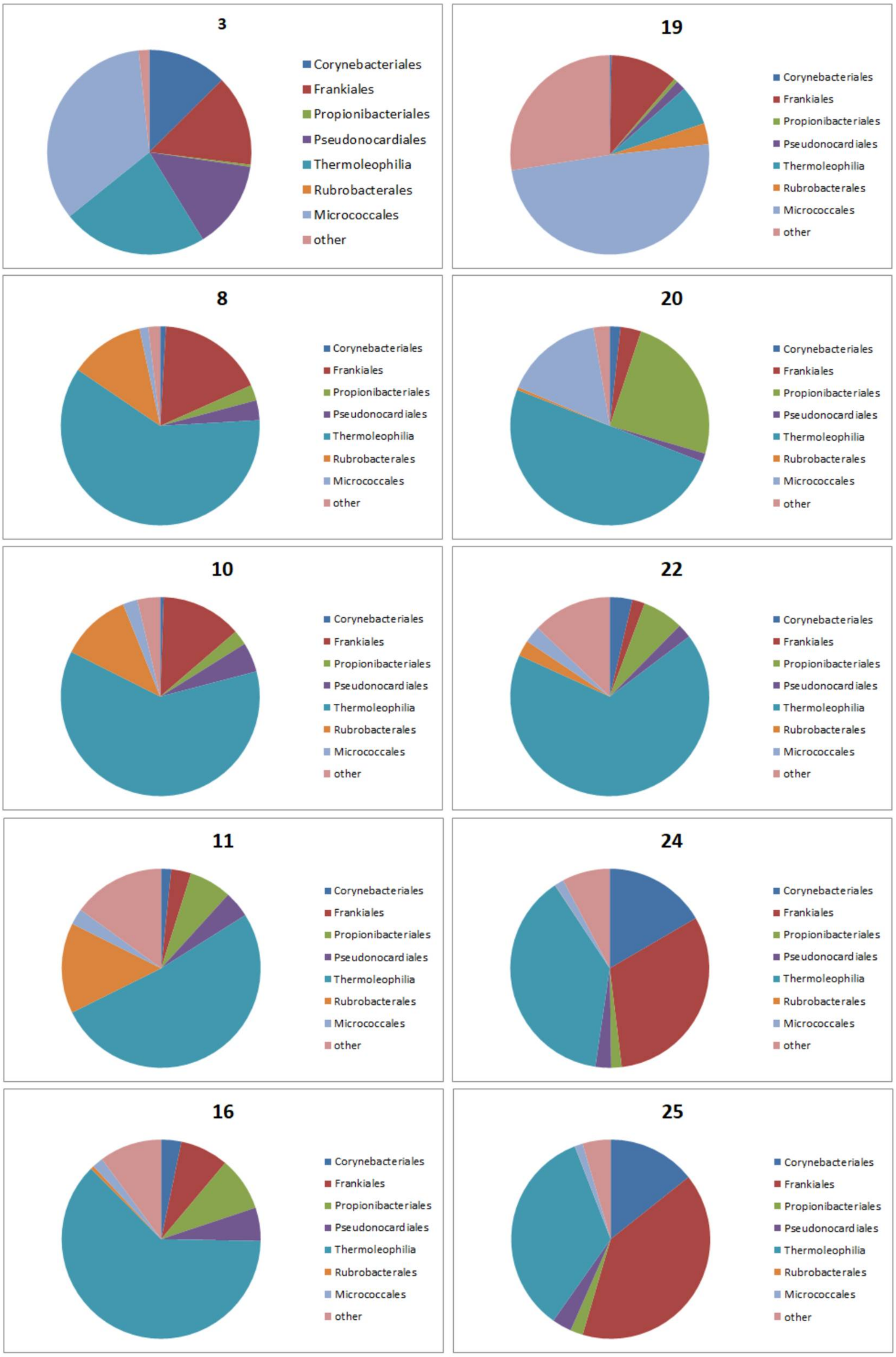

Figure S4. Distribution of selected abundant orders of Actinobacteria (percentage of orders (without Acidimicrobiia) in the class) in 5 selected samples from ancient mining areas (No. 3 Wimmelburg, slag deposit, pH 5.57), 8 (Welfesholz, group C, pH 8.78), 10 (Burgörner, group C, pH 8.85), 11 (Burgörner, group C, pH 8.28), 16 (Uftrungen, Group A, pH 6.23), from industrial mine dump (No. 19, Nienstedt, pH 7.66, extraordinary high electrical conductivity) and four comparative samples (limestone substrate: No. 20 and 22 (group F), acid soil: No. 24, 25 (group G)).

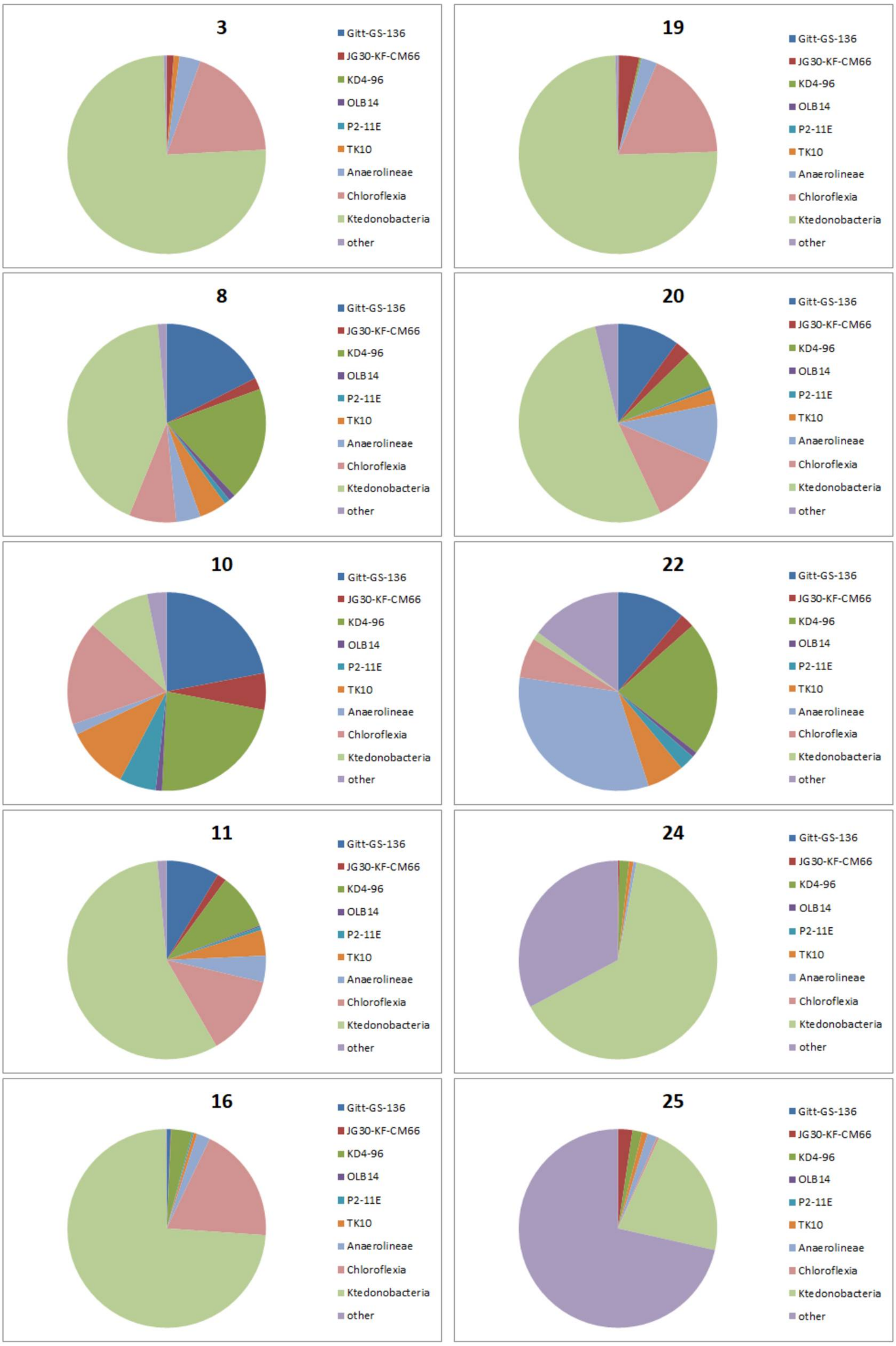

Figure S5. Distribution of abundant OTUs of Chloroflexi (percentage in the class) in 5 selected samples from ancient mining areas (No. 3 Wimmelburg, slag deposit, pH 5.57), 8 (Welfesholz, group C, pH 8.78), 10 (Burgörner, group C, pH 8.85), 11 (Burgörner, group C, pH 8.28), 16 (Uftrungen, Group A, pH 6.23), from industrial mine dump (No. 19, Nienstedt, pH 7.66, extraordinary high electrical conductivity) and four comparative samples (limestone substrate: No. 20 and 22 (group F), acid soil: No. 24, 25 (group G).

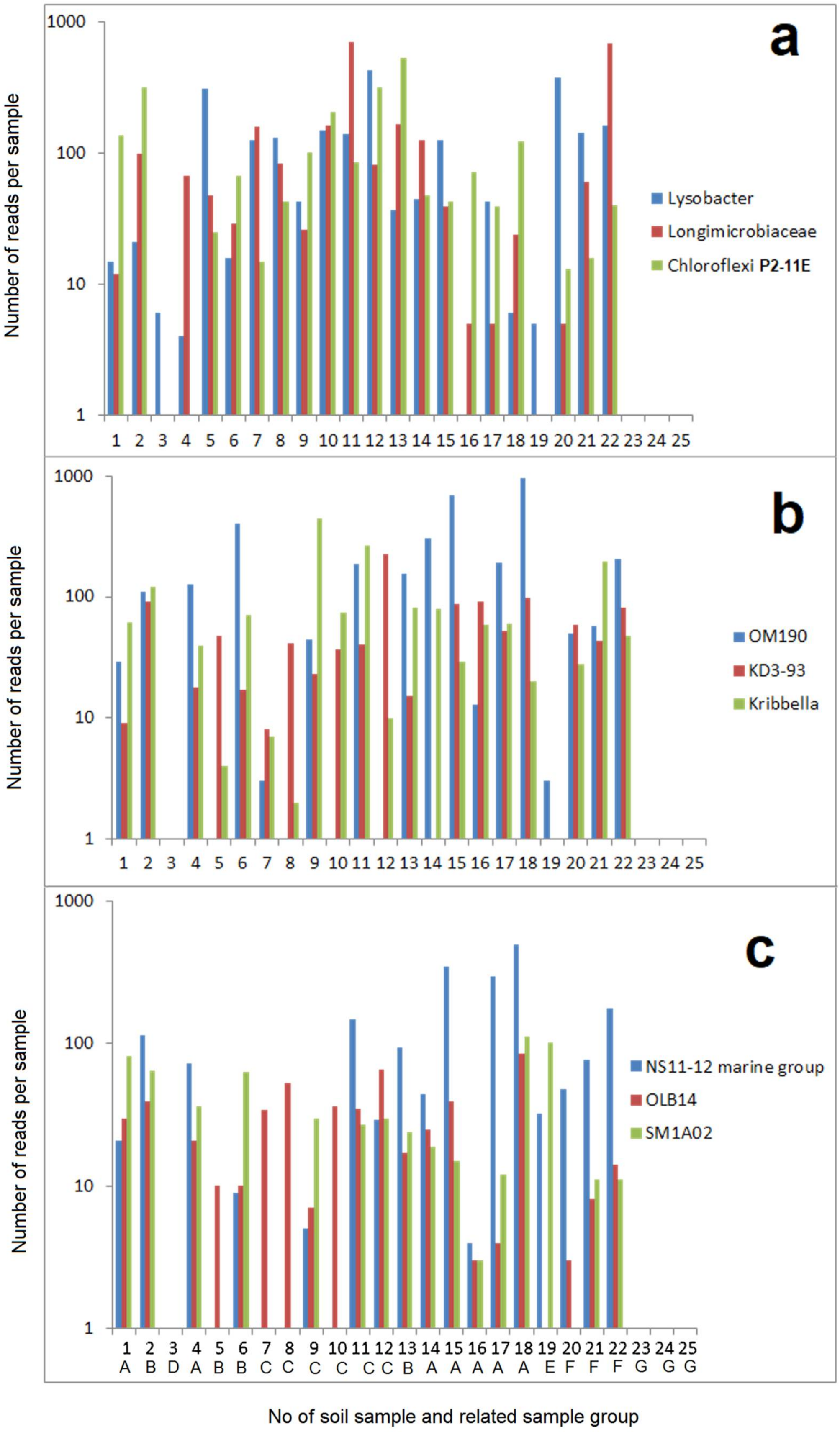

Figure S6. Abundances of nine selected OTUs on ancient mining sites, absent in the comparative samples from acidic Scheme 23–25, group G).

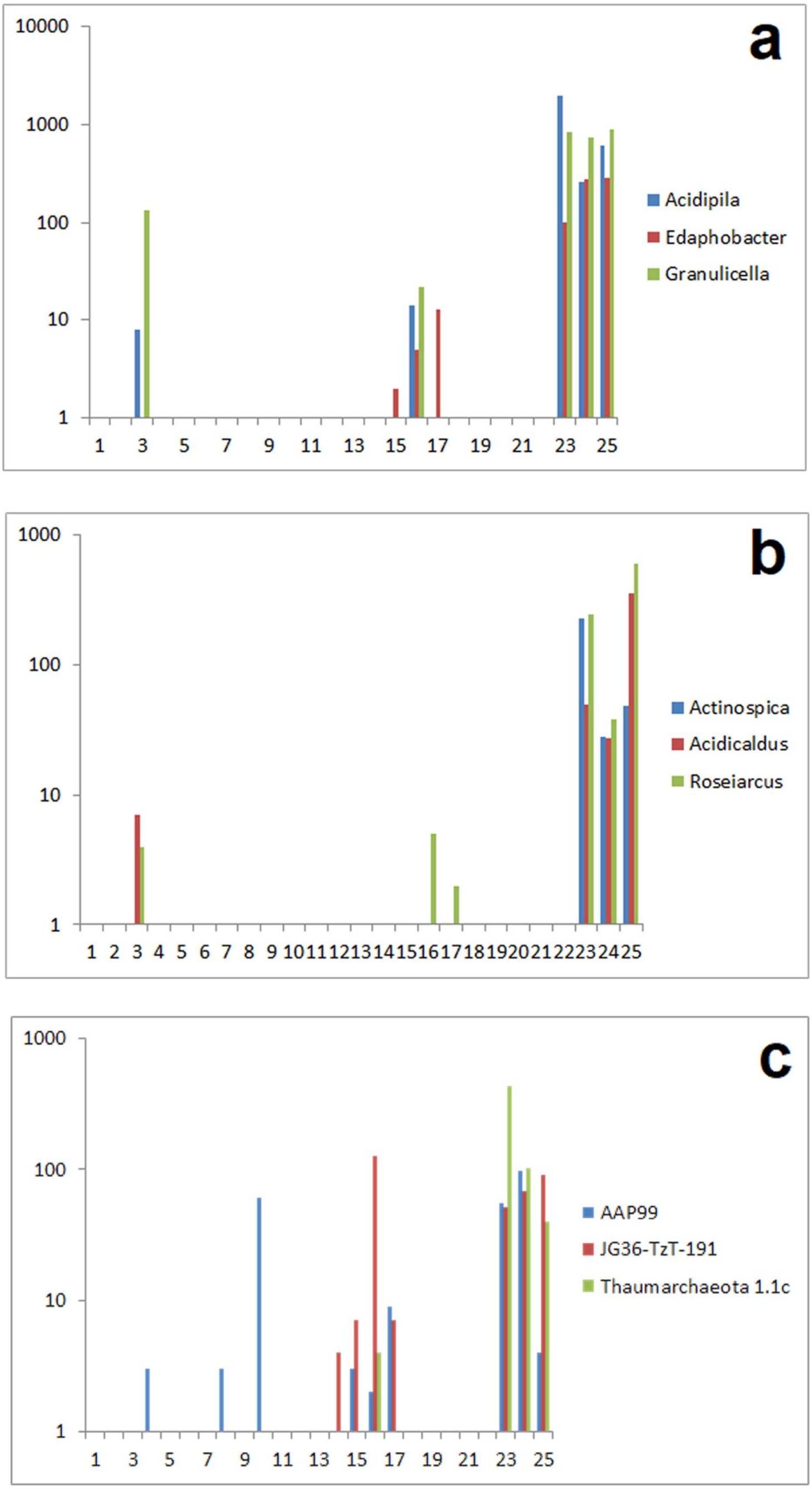

Figure S7. Abundances of nine selected OTUs with acidophilic character and comparatively high representation in the comparative samples from acid soil (No. 23 – 25, group G).

Table S1. Origin of samples.

No.	Location	Group	Gauss-Krüger Coordinates (km)	Internal Lab Designation	pH Value	Electrical Conductivity (µS/cm)
1	Wolferode, pre-industrial mine	A	4466.647/5707.692	E4	7.15	225
2	Wolferode, pre-industrial mine	B	4465.662/5707.686	E6	7.56	220
3	Wimmelburg, slag deposit	D	4466.29/5709.828	E9	5.57	35.4
4	Hergisdorf, pre-industrial mine	A	4463.996/571.,089	E18	7.11	724
5	Wiederstedt, pre-industrial mine	B	4465.778/5726.125	E19	7.72	659
6	Arnstedt, pre-industrial mine	B	4464.656/5727.151	E22	7.63	201
7	Welfesholz, mine dump, early 19th century	C	4470.244/5721.753	E29	8.24	94.5
8	Welfesholz, mine dump, early 19th century	C	4469.068/5721.957	E31	8.78	69.1
9	Burgörner, mine dump, early 19th century	C	4467.222/5721.981	E61	8.27	148
10	Burgörner, mine dump, early 19th century	C	4467.258/5722.002	E63	8.85	82
11	Burgörner, mine dump, early 19th century	C	4467.256/5722.008	E64	8.28	163
12	Burgörner, mine dump, early 19th century	C	4467.32/5722.189	E65	8.01	237
13	Pölsfeld, pre-industrial mine dump	B	4455.803/5710.760	H12	7.69	228
14	Rodishain, pre-industrial mine	A	4424.444/5712.399	Q25	6.98	331
15	Rodishain, pre-industrial mine	A	4424.160/5712.280	Q29	7.04	1240
16	Uftrungen, pre-industrial mine	A	4430.820/5707.821	Q32	6.23	229
17	Uftrungen, pre-industrial mine	A	4431.432/5707.589	Q37	6.79	367
18	Uftrungen, pre-industrial mine	A	4431.725/5707.443	Q42	7.34	344
19	Nienstedt, industrial mine dump	E	4458.609/5699.987	H5	7.66	4677
**Comparative Sites (Limestone Substrate, Forest)**						
20	Burgwenden, Monraburg, prehist. rampart	F	4450.469/5678.312	T72	7.84	183
21	Haynrode, Hasenburg, prehist. rampart, castle	F	3603.354/5702.754	V5	7.82	246
22	Kahla, Dohlenstein, prehist. rampart	F	4472.046/5630.235	B9	6.82	455
**Comparative Sites (Acid Soil, Forest)**						
23	Suhl, Lange Bahn	G	4403.701/5605.545	T51	3.99	56.7
24	Völkershausen, Dietrich	G	3573.264/5627.425	T67	4.01	231
25	Rastenberg, Streitholz	G	4459.745/5672.023	T85	4.42	64.7

Table S2. Groups of soil samples.

Group	Sample Location	pH
A	pre-industrial mines	< 7.5
B	pre-industrial mines	> 7.5
C	mine dump, early 19th century	> 8
D	slag deposit	5.57
E	industrial mine dump	7.66
F	comparative samples from limestone substrate	> 6.5
G	comparative samples from acid soil substrates	< 5

Table S3. Percentage of high abundant OTUs in the samples and reference samples.

		Total	No	1	9	2	16	17	4	6	13	14	15	18	8	10	11	5	7	12	3	19	13	20	21	22	23	24
	sample grp.			A	C	B	A	A	A	B	B	A	A	A	C	C	C	B	C	C	D	E	B	F	F	F	G	G
Ilumatobacteraceae	uncultured	15302		0	0	0	0	0	0	0	0	0	0	0	8	7	0	2	2	0	0	0	0	0	0	0	0	0
Burkholderiaceae	Ralstonia	38788		0	0	1	0	0	0	0	0	0	0	0	21	9	4	0	0	7	0	0	0	0	0	0	0	0
Acidimicrobiia	IMCC26256	33381		1	1	3	1	0	0	1	1	1	0	0	6	9	2	10	2	5	0	0	0	1	1	2	1	1
Sphingomonadaceae	Sphingomonas	25382		3	3	1	1	0	1	2	1	1	0	0	6	5	2	10	8	2	2	0	3	1	1	0	0	1
Chthoniobacteraceae	Candidatus Ud.	60868		2	1	12	26	6	2	7	6	3	1	7	1	1	1	2	2	1	0	0	0	2	2	0	3	0
Pyrinomonadaceae	RB41	58431		0	2	4	8	10	2	2	4	2	4	5	3	6	7	0	1	2	0	0	2	1	8	0	0	0
Acidobacteria	Subgroup 6	51618		4	4	3	2	4	3	6	12	3	6	6	1	1	4	1	3	2	0	0	3	3	4	0	1	0
Nitrososphaeraceae		32410		2	0	5	5	4	3	0	1	1	4	0	0	0	2	0	0	0	1	0	7	6	5	1	2	1
Gaiellales	uncultured	38485		4	6	11	2	1	0	3	4	1	1	0	1	1	3	1	2	1	0	0	5	5	6	0	1	2
Chitinophagaceae	Flavisolibacter	10934		0	0	0	0	0	0	0	0	0	0	0	2	1	1	2	7	1	8	0	3	1	1	0	0	0
Ktedonobacteraceae	uncultured	4863		0	0	0	0	0	0	0	0	0	0	0	0	0	0	0	0	0	21	0	0	0	0	0	0	0
Ktedonobacteraceae	JG30a-KF-32	3978		0	0	0	0	0	0	0	0	0	0	0	0	0	0	0	0	0	15	0	0	0	0	1	0	0
Rubritaleaceae	Luteolibacter	8514		0	0	0	0	0	0	0	0	0	0	0	0	0	0	0	0	0	0	12	0	0	0	0	0	0
Halomonadaceae	Halomonas	4249		0	0	0	0	0	0	0	0	0	0	0	0	0	0	0	0	0	0	7	0	0	0	0	0	0
Marinobacteraceae	Marinobacter	2631		0	0	0	0	0	0	0	0	0	0	0	0	0	0	0	0	0	0	4	0	0	0	0	0	0
Rhodobacteraceae	uncultured	2668		0	0	0	0	0	0	0	0	0	0	0	0	0	0	0	0	0	0	4	0	0	0	0	0	0
Cyclobacteriaceae	Marivirga	1917		0	0	0	0	0	0	0	0	0	0	0	0	0	0	0	0	0	0	3	0	0	0	0	0	0
Flavobacteriaceae	Maribacter	2282		0	0	0	0	0	0	0	0	0	0	0	0	0	0	0	0	0	0	4	0	0	0	0	0	0
Pirellulaceae	Rubripirellula	1607		0	0	0	0	0	0	0	0	0	0	0	0	0	0	0	0	0	0	3	0	0	0	0	0	0

Table S4. Special OTUs from an industrial copper mining deposit with high electrical conductivity (No. 19, Nienstedt, Scheme 5. neither present in soil samples from ancient copper mining places (No. 1–18). Nor in the reference samples (No. 20–25).

Phylum	class	order	family	genus	number of reads
Bacteroidetes	Bacteroidia	Cytophagales	Cyclobacteriaceae	Cyclobacterium	392
Bacteroidetes	Bacteroidia	Cytophagales	Cyclobacteriaceae	Marinoscillum	384
Bacteroidetes	Bacteroidia	Cytophagales	Cyclobacteriaceae	Marivirga	1917
Bacteroidetes	Bacteroidia	Cytophagales	Cyclobacteriaceae	Roseivirga	182
Bacteroidetes	Bacteroidia	Flavobacteriales	Cryomorphaceae	Cryomorpha	185
Bacteroidetes	Bacteroidia	Flavobacteriales	Flavobacteriaceae	Aequorivita	691
Bacteroidetes	Bacteroidia	Flavobacteriales	Flavobacteriaceae	Algibacter	109
Bacteroidetes	Bacteroidia	Flavobacteriales	Flavobacteriaceae	Arcticiflavibacter	656
Bacteroidetes	Bacteroidia	Flavobacteriales	Flavobacteriaceae	Arenibacter	1342
Bacteroidetes	Bacteroidia	Flavobacteriales	Flavobacteriaceae	Cellulophaga	101
Bacteroidetes	Bacteroidia	Flavobacteriales	Flavobacteriaceae	Confluentibacter	312
Bacteroidetes	Bacteroidia	Flavobacteriales	Flavobacteriaceae	Gaetbulibacter	336
Bacteroidetes	Bacteroidia	Flavobacteriales	Flavobacteriaceae	Maribacter	2282
Bacteroidetes	Bacteroidia	Flavobacteriales	Flavobacteriaceae	Muricauda	178
Bacteroidetes	Bacteroidia	Flavobacteriales	Flavobacteriaceae	Pricia	359
Bacteroidetes	Bacteroidia	Flavobacteriales	Flavobacteriaceae	Psychroflexus	626
Bacteroidetes	Bacteroidia	Flavobacteriales	Flavobacteriaceae	Salegentibacter	1416
Bacteroidetes	Bacteroidia	Flavobacteriales	Flavobacteriaceae	Subsaxibacter	764
Bacteroidetes	Bacteroidia	Flavobacteriales	Flavobacteriaceae	Tamlana	861
Bacteroidetes	Bacteroidia	Flavobacteriales	Flavobacteriaceae	uncultured	773
Bacteroidetes	Rhodothermia	Balneolales	Balneolaceae	Balneola	1471
Bacteroidetes	Rhodothermia	Balneolales	Balneolaceae	uncultured	407
Planctomycetes	Planctomycetacia	Pirellulales	Pirellulaceae	Rubripirellula	1607
Proteobacteria	Alphaproteobacteria	Parvibaculales	uncultured		114
Proteobacteria	Alphaproteobacteria	Puniceispirillales	uncultured		110
Proteobacteria	Alphaproteobacteria	Rhizobiales	Rhizobiaceae	Hoeflea	160
Proteobacteria	Alphaproteobacteria	Rhodobacterales	Rhodobacteraceae	Jannaschia	456
Proteobacteria	Alphaproteobacteria	Rhodobacterales	Rhodobacteraceae	Ketogulonicigeniu	707
Proteobacteria	Alphaproteobacteria	Rhodobacterales	Rhodobacteraceae	Loktanella	447
Proteobacteria	Alphaproteobacteria	Rhodovibrionales	Kiloniellaceae	uncultured	213
Proteobacteria	Gammaproteobact.	Cellvibrionales	Cellvibrionaceae	Marinimicrobium	387
Proteobacteria	Gammaproteobac.	Cellvibrionales	Halieaceae	Chromatocurvus	320
Proteobacteria	Gammaproteobact.	Salinisphaerales	Salinisphaeraceae	Salinisphaera	481

Table 5. Selected OTUs with special metabolic and tolerance features.

	samples from ancient mining areas																		reference samples						specific features	ref.
	1	2	3	4	5	6	7	8	9	10	11	12	13	14	15	16	17	18	19	20	21	22	23	24	25		
Tepidiphilus	2	0	0	11	6	0	0	0	0	0	0	12	0	0	0	0	0	0	0	0	0	0	0	0	0	thermophilic	53
Anaerolinea	0	0	0	0	0	0	0	17	0	0	0	0	0	0	1	0	0	0	0	0	0	0	0	0	0	thermophilic	54
Alterococcus	0	0	0	0	0	0	0	0	0	0	0	0	0	8	7	0	27	0	0	0	0	7	0	0	0	thermophilic, agar degradation	55
Brevifollis	0	0	0	11	0	0	0	0	0	0	0	0	1	0	0	0	1	4	0	0	2	0	0	0	0	thermoph, gellan-gum degrad.	56
Pusillimonas	0	0	0	0	0	0	0	0	0	0	1	0	0	1	0	0	0	0	8	0	0	0	0	0	0	salicylate degradation	57
Alkanibacter	0	1	0	1	0	0	0	0	0	0	0	0	1	0	0	2	3	4	0	0	1	0	0	0	0	hexane degradation	58
Povalibacter	0	0	0	0	0	0	0	0	0	0	0	0	0	9	12	0	10	1	0	0	0	0	0	0	0	polyvinylalcohol degradation	59
Immundisolibac.	1	0	0	0	0	2	3	1	0	0	1	29	0	0	2	0	0	1	4	0	0	0	0	0	0	degrad. polycycl. hydrocarbons	60
Fulvivirga	1	0	0	0	0	0	12	0	0	0	1	49	0	0	0	0	0	2	479	0	0	0	0	0	0	salt-tolerant	61
Gulosibacter	0	0	0	0	0	0	0	0	0	0	0	0	0	0	21	0	0	0	0	0	1	0	0	0	0	molinate degradation	62
Tistlia	0	0	0	0	0	0	0	0	0	0	3	0	0	0	0	0	0	0	350	0	0	0	0	0	0	salt-tolerant	63
Lewinella	0	0	0	6	0	0	0	0	0	0	0	0	0	0	25	0	15	2	0	0	0	4	0	0	0	salt-tolerant	64
Marisediminicola	0	15	0	0	1	0	0	0	0	0	0	0	2	0	0	4	1	0	74	0	2	0	0	0	0	salt-tol., special metabolites	65
Salinispora	0	0	0	0	0	0	0	0	0	0	5	0	1	0	0	1	3	1	0	0	0	0	0	0	0	salt-tolerant	66
Halobacteriovrx.	0	0	0	6	0	0	1	0	0	0	0	5	0	0	2	0	0	0	0	0	0	2	0	0	0	salt-tolerant	67

References

1. Rao, M.P.N.; Dong, Z.-Y.; Kann, Y.; Xiao, M.; Kang, Y.Q.; Li, W.-J. *Bacillus tepidiphilus* sp. nov., isolated from tepid spring. *Arch. Microbiol*. **2020** *202*, 2367–2371.
2. Yamada, T.; Skigichi, Y.; Hanada, S.; Imachi, H.; Ohashi, A.; Harada, H.; Kamagata, Y. *Anaerolinea thermolimosa* sp. Nov., *Levilinea saccharolytica* gen. nov., sp. Nov. and *Leptolinea tardivitalis* gen. nov., sp. Nov., novel filamentous anaerobes, and description of the new classes *Anaerolineae classis* nov. and *Caldilineae classis* nov. in the bacterial phylum Chloroflexi. *Int. J. Syst. Evol. Microbiol.***2006**, *56*, 1843–1851.
3. Shieh, W.Y.; Jean, W.D. *Alterococcus agarolyticus*, gen. nov., sp. nov., a halophilic thermophilic bacterium capable of agar degradation. *Can. J. Microbiol.* **1998**, *44*, 637–645.
4. Otsuka, S.; Suenaga, T.; Vu, H.T.; Ueda, H.; Yokota, A.; Senoo, K. *Brevifollis gellanilyticus* gen. nov., sp. nov., a gellan-gum-degrading bacterium of the phylum *Verrucomicrobia*. *Int. J. Syst. Evol. Microbiol.* **2013**, *63*, 3075–3078.
5. Park, M.S.; Park, Y.-J.; Jung, J.Y.; Lee, S.H.; Park, W.; Lee, K.; Jeon, C.O. *Pusillimonas harenae* sp. nov., isolated from sandy beach, and emended description of the genus *Pusillimonas*. *Int. J. Syst. Evol. Microbiol.* **2011**, *61*, 2901–2906.
6. Friedrich, M.M.; Lipski, A. *Alkanibacter difficilis* gen. nov., sp. nov. And *Singularimonas variicoloris* gen. nov., sp. nov., hexane-degrading bacteria isolated from a hexane-treated biofilter. *Int. J. Syst. Evol. Microbiol.* **2008**, *58*, 2324–2329.
7. Nogi, Y.; Yoshizumi, M.; Hamana, K.; Miyazaki, M.; Horikoshi, K. *Povalibacter uvarum* gen. nov., sp. nov., a polyvinyllalcohol-degrading bacterium isolated from grapes. *Int. J. Syst. Evol. Microbiol.* **2014**, *64 Pt 8*, 2712–2717.
8. Corteselli, E.M.; Aitken, M.D.; Singleton, D.R. Description of *Immundisoblibacter cernigliae* gen. nov., sp. nov., a high-molecular-weight polycyclic aromatic hydrocarbon-degrading bacterium within the class *Gammaproteobacteria*, and proposal of *Immundisolibacterales ord*. nov. and *Immundisolibacteraceae fam*. nov. *Int. J. Syst. Evol. Microbiol.* **2017**, *67*, 925–931.
9. Nedashkovskaya, O.I.; Kim, S.B.; Shin, D.S.; Beleneva, I.A.; Mikhailov, V.V. *Fulvivirga kasyanovii* gen. nov., sp. nov., a novel member of the phylum *Bacteroidetes* isolated from seawater in a mussel farm. *Int. J. Syst. Evol. Microbiol.***2007**, *57*, 1046–1049.
10. Manaia, C.M.; Nogales, B.; Weiss, N.; Nunes, O.C. *Gulosibacter molinativorax* gen. nov., sp. nov., a molinate- degrading bacterium, and classification of *′Brevibacterium helvolum*'DSM 20419 as *Pseudoclavibacter helvolus* gen. nov., sp. nov. *Int. J. Syst. Evol. Microbiol.* **2004**, *54*, 783–789.
11. Diaz-Cárdenas, C.; Patel, B.-K.C.; Baena, S. Tistlia consotensis gen. nov., sp. nov., an aerobis, chemoheterotrophic, free-living, nitrogen-fixing alphaproteobacterium, isolated from a Colombian saline spring. *Int. J. Syst. Evol. Microbiol.* **2010**, *60*, 1437–1443.
12. Khan, S.T.; Fukunaga, Y.; Nakagawa, Y.; Harayama, S. Emended descriptions of the genus *Lewinella* and of *Lewinella chaerens*, *Lewinella nigricans* and *Lewinella persica*, and descriptions of *Lewinella lutea* sp. nov. and Lewinella marina sp. nov. *Int. J. Syst. Evol. Microbiol.* **2007**, *57*, 2946–2951.
13. Li, H.-R.; Yu, Y.; Luo, W.; Zeng, Y.-X. *Marisediminicola antarctica* gen. nov., sp. nov., an actinobacterium isolated from the Antarctic. *Int. J. Syst. Evol. Microbiol.* **2010**, *60*, 2535–2539.
14. Jensen, P.R.; Moore, B.S.; Fenical, W. The Marine Actinomycete Genus Salinispora: A Model Organism for Secondary Metabolite Discovery. *Nat. Prod. Rep.* **2015**, *32*, 738–751.
15. Ottaviani, D.; Chierichetti, S.; Angelico, G.; Forte, C.; Rocchegiani, E.; Manuali, E.; Leoni, F. *Halobacteriovorax* isolated from marine water of the Adriatic Sea, Italy, as an effective predator of *Vibrio parahaemolyticus*, non-01/0139 V. cholerae, V. vulnuficus. *J. Appl. Microbiol.* **2018**, *125*, 1199–1207.
